# Supplementary material for: Identification of photoperiod-induced specific miRNAs in the adrenal glands of Sunite sheep (Ovis aries)
Source: Front Vet Sci. 2022 Jul 22;9:888207. doi: 10.3389/fvets.2022.888207 (PMC9354845; doi:10.3389/fvets.2022.888207)
Supplement: Supplementary file 1 [file Data_Sheet_1.ZIP › Supplementary file/miRDeep2 file.zip!/miRDeep2 file/Novel_Structure/SPLP42A1/Novel_561.pdf]

Provisional ID	:	NC_019459.2_3314
Score total	:	2.6
Score for star read(s)	:	-1.3
Score for read counts	:	0
Score for mfe	:	2.3
Score for randfold	:	1.6
Score for cons. seed	:	
Total read count	:	227
Mature read count	:	227
Loop read count	:	0
Star read count	:	0

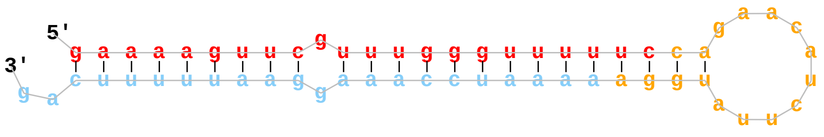

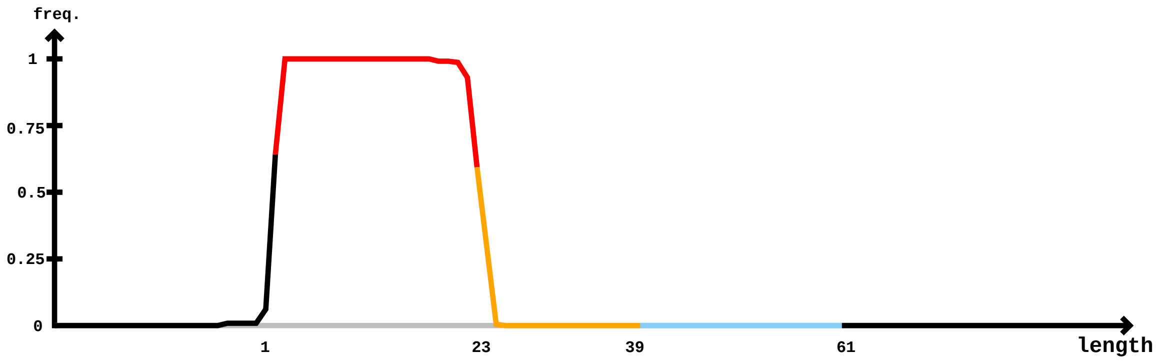

Mature **Star**

```
5'- ucaaauaauaauuggguuggcugaaaaguucguuuggguuuuuccagaacaucuuauggaaaaauccaaaggaauuuuucagccaacacaauauuaacuuucacagcuuug   -3'    exp
    .....((((((((.((((((((((((((((.((((((((((((((..........)))))))))))))).))))))))))))))).))))))))................    reads     mm        sample
    .................uggcugaaaaguucAuuugggu........................................................................    2         1         seq
    .....................ugaaaaguucguuuggguuu......................................................................    1         0         seq
    .....................ugaaaaguucguuuggguuuu.....................................................................    2         0         seq
    .....................ugaaaaguucguuuggguuuuu....................................................................    9         0         seq
    ......................gaaaaguucguuuggguuuuu....................................................................    5         0         seq
    ......................Aaaaaguucguuuggguuuuu....................................................................    62        1         seq
    ......................Aaaaaguucguuuggguuuuuc...................................................................    66        1         seq
    .......................aaaaguucguuuggguuuC.....................................................................    1         1         seq
    .......................aaaaguucguuuggguuuu.....................................................................    10        0         seq
    .......................aaaaguucguuuggguuuuG....................................................................    1         1         seq
    .......................aaaaguucguuuggguuuuuA...................................................................    3         1         seq
    .......................aaGaguucguuuggguuuuuc...................................................................    2         1         seq
    .......................aaaGguucguuuggguuuuuc...................................................................    1         1         seq
    .......................aaaaguucguuuggguuuuucA..................................................................    56        1         seq
    .......................aaaaguucguuuggguuuuucG..................................................................    7         1         seq
    .......................aaaaguucguuuggguuuuucAa.................................................................    1         1         seq
```
